# Safety and Efficacy of Enfortumab Vedotin in a Patient With Metastatic Urothelial Carcinoma on Dialysis: A Case Report

**DOI:** 10.7759/cureus.77948

**Published:** 2025-01-25

**Authors:** Yuto Tsubonuma, Yujiro Nagata, Katsuyoshi Higashijima, Akinori Minato, Ikko Tomisaki, Naohiro Fujimoto

**Affiliations:** 1 Urology, University of Occupational and Environmental Health, Kitakyushu, JPN; 2 Urology, Kurate Hospital, Kurate, JPN

**Keywords:** complete response, enfortumab vedotin, hemodialysis, metastatic urothelial carcinoma, renal failure

## Abstract

Recently, enfortumab vedotin has emerged as a promising treatment option for metastatic urothelial carcinoma. Although it does not require dosage adjustments in patients with renal failure, its safety and efficacy, particularly in those undergoing dialysis, remain unclear. We report a case of metastatic urothelial carcinoma in a patient undergoing hemodialysis who achieved a complete response to enfortumab vedotin therapy without severe adverse events. This complete response was maintained for two years without any severe complications. Our case demonstrates that enfortumab vedotin can be safely administered long-term to patients undergoing hemodialysis.

## Introduction

Patients with metastatic urothelial carcinoma (mUC) have a poor prognosis, with a relatively low five-year survival rate (8.3% for bladder cancer) [1]. Until recently, platinum-based chemotherapy was the first-line standard therapy for mUC. For gemcitabine/cisplatin (GC), one of the standard platinum-based chemotherapy, median progression-free survival (mPFS) was 7.7 months and the objective response rate (ORR) was 49.4% in a phase 3 trial [2].

Avelumab maintenance therapy is recommended for patients with mUC not progressed following platinum-based chemotherapy. In a phase 3 trial (JAVELIN Bladder 100), avelumab maintenance therapy improved mPFS (3.7 months vs. 2.0 months) and ORR (9.7% vs. 1.4%) compared with best supportive care [3]. Recently, enfortumab vedotin (EV) has become the standard therapy after platinum-based chemotherapy and disease progression during or after treatment with an immune checkpoint inhibitor for mUC, with a phase 3 clinical trial (EV-301) providing strong evidence to support its efficacy and safety [4,5]. In EV-301, EV showed improvements in mPFS (5.6 months vs. 3.7 months) and ORR (40.6% vs. 17.9%) compared with chemotherapy [5]. Furthermore, since 2023 in the United States, and 2024 in the European Union and Japan, EV plus pembrolizumab has been approved as the first-line therapy for mUC. In a phase 3 trial (EV-302), EV plus pembrolizumab demonstrated better mPFS (12.5 months vs. 6.3 months) and ORR (67.7% vs. 44.4%) compared with platinum-based chemotherapy [6].

Although EV is a key drug for mUC, there are few case reports demonstrating the use of EV for short periods in patients with mUC on dialysis patients, and patients on dialysis have not been included in large-scale clinical trials on EV [4-7]. Thus, the effect of dialysis on EV pharmacokinetics remains unknown. Here, we report a case of a patient who safely achieved a complete response to EV therapy for two years despite undergoing hemodialysis.

## Case presentation

A 41-year-old male patient was referred to our hospital with a diagnosis of bladder cancer (pT2, urothelial carcinoma (UC), high-grade) treated with transurethral resection of the bladder tumor. He had a history of smoking 40 cigarettes per day for 12 years. There was no family history of cancer in his first-degree relatives or exposure to occupational carcinogens. He did not have any comorbidities. Three months after diagnosis, he underwent radical cystectomy with neobladder reconstruction. Thirty-six months later, he experienced a recurrence of bilateral urinary tract UC (pT2, high-grade), leading to bilateral radical nephroureterectomy and the neobladder resection with simultaneous initiation of hemodialysis.

**Figure 1:**
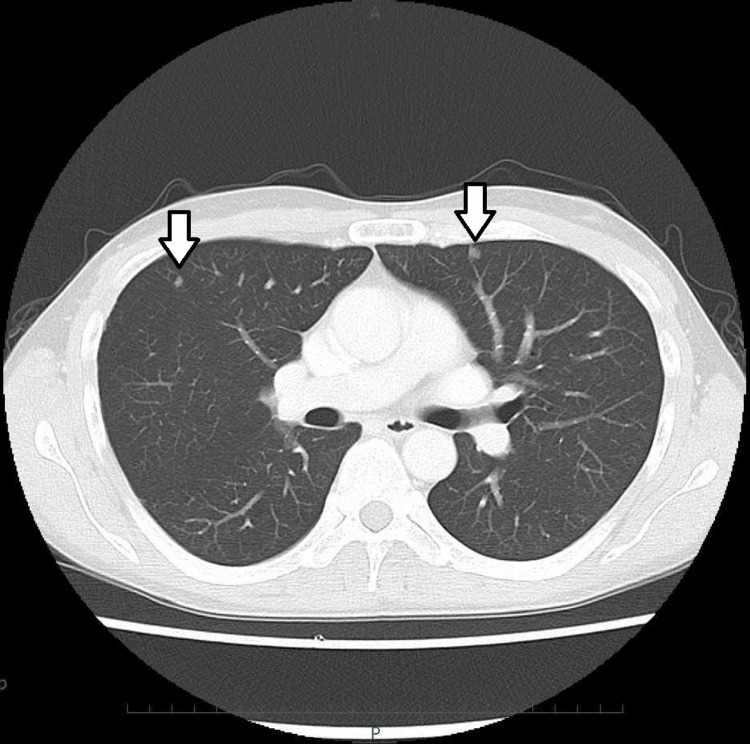
CT scan prior to EV treatment showing two lesions in the lung (white arrows). EV: enfortumab vedotin

Sixty-three months after bilateral radical nephroureterectomy, chest computed tomography (CT) revealed multiple pulmonary metastases. There were a total of eight lung metastases, including one lesion measuring 8 mm, three lesions measuring 6 mm, and four lesions measuring 4 mm in size. No biopsy or programmed cell death ligand 1 evaluation was performed. First-line treatment with GC was initiated, which resulted in a partial response after two cycles, followed by the administration of avelumab as a first-line maintenance treatment. Unfortunately, CT evaluation after seven cycles of avelumab showed two new lung metastases. The size of the lung metastases was 9 mm and 8 mm (Figure 1). His body mass index was 19 kg/m^2^ and his Eastern Cooperative Oncology Group (ECOG) performance status was 0.

**Figure 2:**
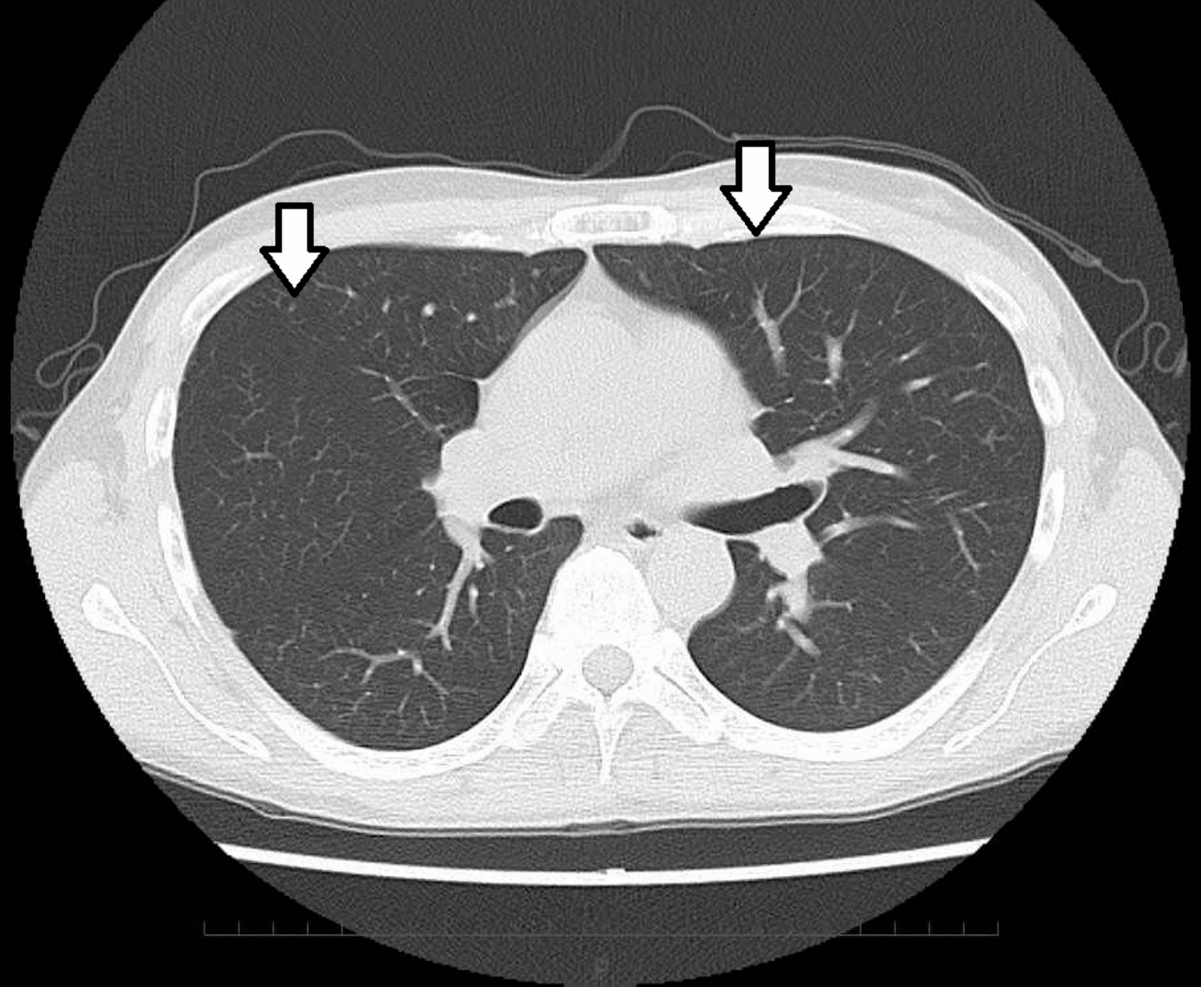
CT scan at first follow-up after two cycles of EV showing complete response with resolution of all lesions (white arrows). EV: enfortumab vedotin

After disease progression, the patient received second-line therapy with EV at 1.25 mg/kg (full dose) administered on days 1, 8, and 15 of a 28-day cycle. Hemodialysis was performed approximately 24 hours after EV infusion, and no electrolyte abnormalities requiring emergency hemodialysis were observed during the treatment period. According to Common Terminology Criteria for Adverse Events (CTCAE) v.5.0 [8], he experienced a grade 2 rash seen on the right medial upper arm, pubic region, and thighs as an adverse event during the first cycle, which was manageable with a dose reduction (1.00 mg/kg) of EV and an antihistamine. After two cycles of EV treatment, CT revealed a complete response (Figure 2). 

**Figure 3:**
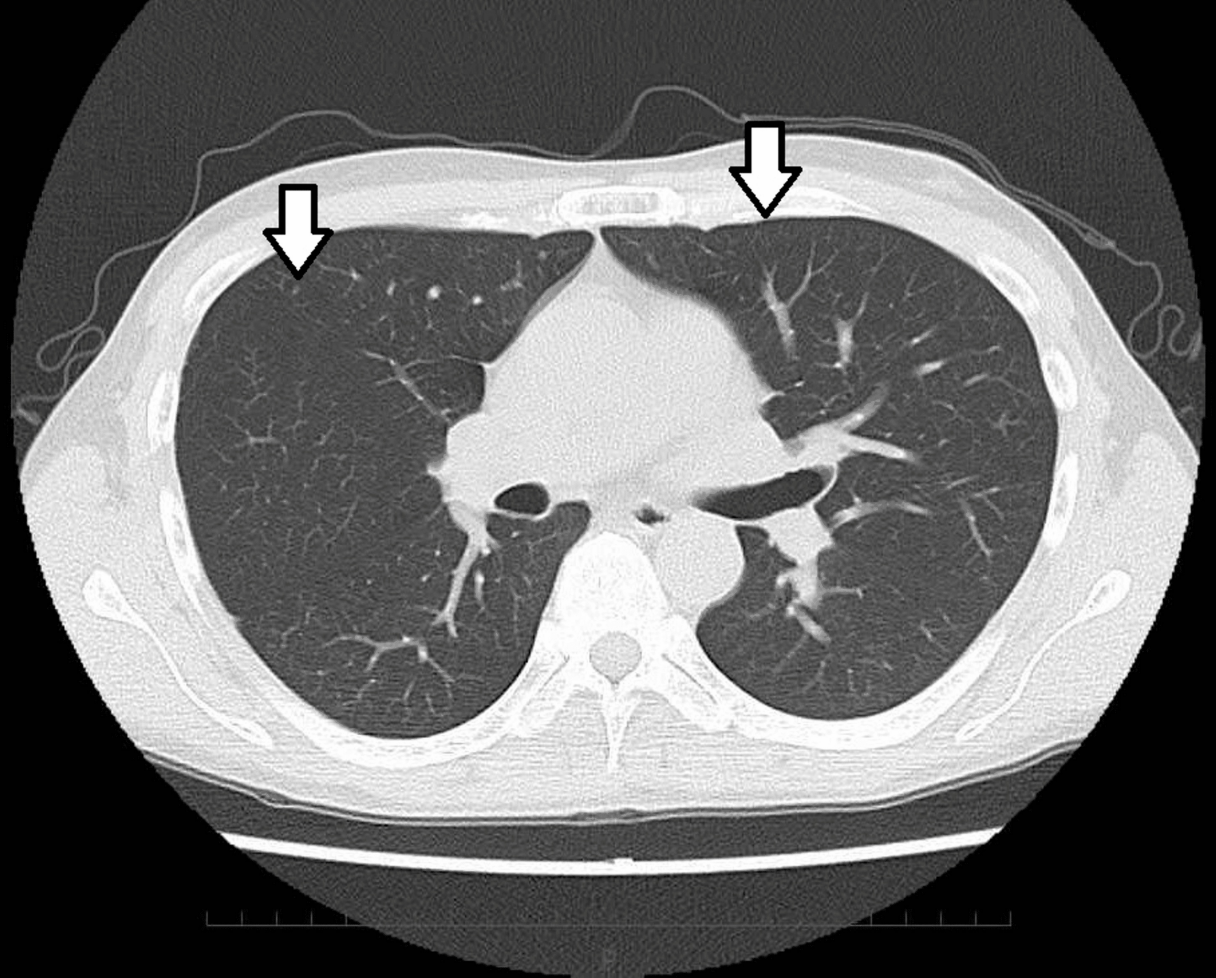
CT scan at follow-up two years after initiation of EV showing complete response with the resolution of all lesions (white arrows). EV: enfortumab vedotin

During the sixth cycle of EV, he developed grade 2 fatigue following the day 8 administration; consequently, the day 15 dose was not administered. After a two-week break, the fatigue resolved, and the day 15 dose was omitted from the subsequent courses. He then maintained a complete response for two years without experiencing any severe adverse events (Figure 3).

## Discussion

EV is a monomethyl auristatin E (MMAE)-containing antibody-drug conjugate against nectin-4, a highly expressed protein in UC [9]. MMAE is a microtubule inhibitor, that inhibits cell proliferation and induces cell death [10]. The catabolism of EV in humans has not been fully studied. However, EV is believed to be cleaved by proteolytic enzymes, releasing MMAE [11], which is primarily metabolized by CYP3A4 in the liver [12]. In a study on brentuximab vedotin, another antibody-drug conjugate containing MMAE, 17% of the total administered MMAE was recovered in feces and 6% in urine, mostly in unchanged forms [12], and similar outcomes were expected for EV. In the EV-301 study, 136 of 301 patients had moderate renal dysfunction (creatinine clearance (CrCL) 30-60 mL/minute), and four had severe renal dysfunction (CrCL <30 mL/minute) [5]. Notably, no significant differences were observed in EV and MMAE exposure between patients with renal dysfunction and those with normal renal function [9]. These findings suggest that the primary elimination pathways for MMAE are hepatic metabolism and biliary excretion, implying that EV therapy can be safely administered to patients with severe renal failure.

Owing to the large molecular weight of EV (approximately 152 kDa) and the relatively high in vitro binding of MMAE to plasma proteins (68-82%) [9], EV was not removed by dialysis. Therefore, EV exposure was not affected by dialysis, allowing for full-dose administration in this case.

Few studies have examined the use of EV in patients with mUC on dialysis, with all reporting the administration of EV at a full dose (Table 1). Isoda et al. reported CR after two cycles of EV with grade 1 adverse events [13]. Similarly, Collette et al. reported a case in which near-CR was observed after three EV courses in a patient undergoing peritoneal dialysis [14]. Adverse events included rash (grade 2), which was treated with oral steroids and antihistamines. Mori et al. reported two cases of EV treatment in patients undergoing dialysis [15]. In the first case, a partial response was achieved after two cycles of EV, with manageable neutropenia (grade 3), rash (grade 2), and dysgeusia (grade 2). In the second case, progressive disease occurred after two cycles of EV accompanied by rash (grade 2), which was treated with oral steroids. These reports indicate that EV can be safely administered for short periods in patients undergoing dialysis. However, to the best of our knowledge, no studies have assessed the safety and efficacy of long-term EV treatment in patients on dialysis for ≥ 2 years.

**Table 1:** Summary of dialysis patients with metastatic urothelial carcinoma who received enfortumab vedotin. AE: adverse event; CR: complete response; PFS: progression-free survival; PD: progressive disease; PR: partial response.

Authors, Year	Types of dialysis	Duration (months) of EV treatment	Metastatic lesions	Best response	PFS (months)	AE grade 1-2	AE grade 3-4
Current case	Hemodialysis	24	Lung	CR	N/A	Rash, fatigue	No
Isoda et al., 2023 [13]	Hemodialysis	3	Lung	CR	N/A	Alopecia, dysgeusia	No
Collette et al., 2022 [14]	Peritoneal dialysis	≥3	Lung, bone	Near CR	N/A	Rash	No
Mori et al., 2024 [15]	Hemodialysis	4	Peritoneal dissemination	PR	4	Rash, dysgeusia	Neutropenia
Mori et al., 2024 [15]	Hemodialysis	2	Para-aortic lymph node	PD	2	Rash, pruritus	No

A long-term analysis of the EV-301 study, with a median follow-up of two years, identified treatment-related skin reactions (14.9%), peripheral neuropathy (5.1%), and fatigue (6.8%) as the most common grade 3 or higher adverse events associated with EV [4]. Similarly, a European multicenter cohort study of patients with UC treated with EV reported peripheral neuropathy as the most frequent grade 3 or higher adverse event, occurring in 9.6% of patients [16]. The median time to onset of peripheral neuropathy was 2.81 months (interquartile range: 0.03-13.04 months) [4]. Peripheral neuropathy was the most common cause of discontinuation during the early stages of EV administration.

The current patient did not have any grade 3 or higher adverse events but only grade 2 skin rash or fatigue. In the long-term analysis of the EV-301 study, treatment-related skin reactions and fatigue accounted for a high percentage of grade 2 or lower adverse events occurring in 32.4% and 24.6% of patients, respectively [4]. Our patient demonstrates the safety of EV therapy in patients undergoing dialysis, as the patient was able to continue treatment for two years without any serious adverse events, which are often a cause of discontinuation in patients not undergoing dialysis.

## Conclusions

The limited number of studies on EV use in hemodialysis patients, including ours, suggests that its effectiveness and safety are not yet fully established. However, this case report indicates that EV can be administered to patients undergoing hemodialysis for an extended period without significant safety concerns. As more antibody-drug conjugates are approved and used more frequently in patients on dialysis, further studies are needed to validate the safety of antibody-drug conjugates in these patients.
